# Comprehensive analysis identifies crucial genes associated with immune cells mediating progression of carotid atherosclerotic plaque

**DOI:** 10.18632/aging.205566

**Published:** 2024-02-20

**Authors:** Zhen Li, Junhui Liu, Zhichun Liu, Xiaonan Zhu, Rongxin Geng, Rui Ding, Haitao Xu, Shulan Huang

**Affiliations:** 1 Department of Neurosurgery III, Renmin Hospital of Wuhan University, Wuhan 430060, Hubei Province, P.R. China

**Keywords:** carotid atherosclerotic plaque, bioinformatics, RNA sequencing, WGCNA, immunity

## Abstract

Backgrounds: Carotid atherosclerosis is prone to rupture and cause ischemic stroke in advanced stages of development. Our research aims to provide markers for the progression of atherosclerosis and potential targets for its treatment.

Methods: We performed a thorough analysis using various techniques including DEGs, GO/KEGG, xCell, WGCNA, GSEA, and other methods. The gene expression omnibus datasets GSE28829 and GSE43292 were utilized for this comprehensive analysis. The validation datasets employed in this study consisted of GSE41571 and GSE120521 datasets. Finally, we validated PLEK by immunohistochemistry staining in clinical samples.

Results: Using the WGCNA technique, we discovered 636 differentially expressed genes (DEGs) and obtained 12 co-expression modules. Additionally, we discovered two modules that were specifically associated with atherosclerotic plaque. A total of 330 genes that were both present in DEGs and WGCNA results were used to create a protein-protein network in Cytoscape. We used four different algorithms to get the top 10 genes and finally got 6 overlapped genes (TYROBP, ITGB2, ITGAM, PLEK, LCP2, CD86), which are identified by GSE41571 and GSE120521 datasets. Interestingly, the area under curves (AUC) of PLEK is 0.833. Besides, we found PLEK is strongly positively correlated with most lymphocytes and myeloid cells, especially monocytes and macrophages, and negatively correlated with most stromal cells (e.g, neurons, myocytes, and fibroblasts). The expression of PLEK were consistent with the immunohistochemistry results.

Conclusions: Six genes (TYROBP, ITGB2, ITGAM, PLEK, LCP2, CD86) were found to be connected with carotid atherosclerotic plaques and PLEK may be an important biomarker and a potential therapeutic target.

## INTRODUCTION

Atherosclerosis is a chronic progressive inflammation-related disease that occurs in the arteries and is characterized by the formation of plaques on damaged artery walls. It can occur in a variety of arteries throughout the body, most commonly the coronary, carotid, and peripheral arteries. In comparison to early plaques, advanced carotid atherosclerotic plaque has a higher propensity to rupture, resulting in transient ischemic attack or ischemic stroke, making it a significant contributor to global mortality.

Various applications have utilized data mining, such as sequencing [1], analysis of gene expression using microarrays [2–4], detection of single-nucleotide polymorphisms [5, 6], and more. WGCNA, a robust technique, can be employed to detect gene co-expression modules, investigate the relationship between the modules and phenotypes, and uncover pivotal genes that govern crucial biological mechanisms [7]. xCell is an innovative approach that utilizes gene signatures to detect 64 different types of immune and stromal cells through comprehensive *in silico* investigations [8]. This method allows accurate calculation of the relative proportions of immune and stromal cell composition in a lesion sample.

Despite the fact that research has shown that persistent inflammation can contribute to the development of atherosclerosis, the precise mechanism behind atherosclerosis remains unknown. Moreover, there have been fewer investigations carried out to examine the association between genes and immune cells in big data related to atherosclerosis.

To examine the immunocyte infiltration microenvironment and determine the important genes linked to carotid atherosclerotic plaque, we employed GSE28829 and GSE43292 as the training datasets for this investigation. The validated hub genes were then further confirmed in GSE41571 and GSE120521, as well as through immunohistochemistry (IHC) analysis of clinical samples.

## MATERIALS AND METHODS

### Data source

GSE28829 [9] and GSE43292 [10] were downloaded from the Gene Expression Omnibus (GEO) database [11] (https://www.ncbi.nlm.nih.gov/geo/). The GSE28829 profile includes 16 advanced atherosclerotic plaques and 13 early atherosclerotic plaques on the GPL570 [HG-U133_Plus_2] Affymetrix Human Genome U133 Plus 2.0 Array. The GSE43292 dataset included 32 atheroma plaques and 32 macroscopically intact tissues on the GPL6244 platform, specifically using the HuGene-1_0-st Affymetrix Human Gene 1.0 ST Array in its transcript (gene) version. The training dataset was identified as GSE28829 and GSE43292.

The Department of Pathology, People's Hospital of Wuhan University provided 23 carotid atherosclerotic plaques and 11 carotid intact tissues. These samples were collected between 2017 and 2022.

### Data preprocessing and DEG screening

Screening and preprocessing of data for differential expression gene (DEG) analysis, the series matrix files of GES28829 and GSE43292 were annotated using data tables from GPL570 and GPL6244, respectively. This annotation involved replacing the probe name with the official gene symbol to obtain the gene expression matrix. Then, the gene expression matrix of GSE28829 and GSE43292 were combined in RStudio [12–14]. The “sva” R package was used to remove batch effects. Besides, the screening of differentially expressed genes (DEGs) was performed using “limma” R package. DEG screening was performed using thresholds of |Fc (Fold-change)|>1.5 and adjusted P<0.05. Significantly regulated transcripts and genes were visualized in a volcano plot created using the ggplot2 package in the R software. With the “pheatmap” package, the top 50 upregulated and downregulated genes were visualized.

### Functional enrichment analysis

The above up- and down-regulated genes were imported into the Database for Annotation, Visualization, and Integrated Discovery (DAVID, v6.8) [15], official gene symbols were selected as identifiers, and Homo sapiens was selected as a species. Finally, the role of genes and gene products in any organism was obtained from Gene Ontology (GO) [16] and metabolic pathways and gene signaling networks were obtained according to Kyoto Encyclopedia of Genes and Genomes (KEGG) [17]. This study shows the top five results sorted by P-value (P<0.05) from high to low.

### Gene set enrichment analysis

GSEA analysis was performed on the above dataset using GSEA.4.2.3 [18] software. Input plaque grouping data and select c2.cp.kegg.v2023.1.Hs.symbols.gmt to assess the associated pathways and molecular mechanisms. The minimum gene set was set to be 5, the maximum gene set to be 5000, and 1000 were resampled. The screening criteria included a normalized enrichment score>1.5 and a false discovery rate (FDR)<0.01.

### Co-expression network construction by WGCNA

We used Weighted Gene Co-Expression Network Analysis (WGCNA) built with the R package “WGCNA” [19] to identify co-expression networks. We used gene expression profiles to calculate the MAD (median absolute deviation) of each gene, removing the top 50% of genes with the smallest MAD. Outliers and samples were removed using the GoodSamplesGenes method in the R package WGCNA, and scale-free co-expression networks were further constructed using WGCNA.

The pickSoftThreshold function was used to choose the soft threshold power(β) based on the criterion of scale-free topology. Afterwards, the gene co-expression modules were discovered utilizing the method of constructing networks in a single step. Every module contained at least 30 genes. By utilizing the dynamic cutting tree technique, the threshold was established at 0.25.

### Selecting important modules

Module genes (ME) of every module were calculated using the R ggcorr software package and correlations within the ME between modules were shown. Based on the moduleTraitCor and moduleTraitPvalue algorithms, the two modules with significant correlation (P<0.05) with plaque features and the strongest positive and negative correlations were assumed to be the key modules for further analysis. Genes with module membership greater than 0.8 (MM, ME correlation with gene expression profile) were screened for further analysis.

### Functional enrichment analysis on selected modules

After the aforementioned screening, the genes within each module were utilized for conducting BP and KEGG enrichment analyses. After adjusting the P-value, the first 10 terms of BP enrichment analysis and the first 9 terms of KEGG enrichment analysis were visualized.

### PPI network construction and screening for key genes

After intersecting the differentially expressed genes (DEGs), we proceeded to identify the genes within the modules. Next, the intersecting genes were submitted to the STRING [20] (version 11.5) platform to determine the protein-protein interactions, with a minimum interaction score of 0.6. Then, we imported the obtained network into Cytoscape software [21] for visualization. In addition, we applied the cytohubba plugin [22] to identify key genes by Degree algorithm. The top 10 genes were filtered from the Degree algorithm. Then, we intersected the top 10 genes from 4 different algorithms and obtained 6 hub genes.

### Validation of hub genes

Six genes were screened from the four algorithms. Then, GSE41571 and GSE120521 were combined to validate genes that may be involved in carotid atherosclerotic plaque progression. The diagnostic and discriminatory value of the six hub genes in the plaque and intact tissue groups was assessed using ROC curve [23].

### Immune cell infiltration analysis

To understand the immune landscapes of the genetic profile of advanced atherosclerotic plaques, we used a new method based on genetic characterization, xCell, to identify 64 cell types by extensive *in silico* analysis. The 64 different cell types were classified into lymphoid, myeloid, stromal, stem cells, and additional categories. Significant immune cells were then identified between plaque and intact tissue samples using the Wilcoxon threshold test [24] at P < 0.05.

### Relevance analysis of genes and immune cells

The relationship between the expression of the six pivotal genes and the relative proportion of immune cells in plaque and intact tissue samples was illustrated by Spearman's correlation test analysis, respectively. Correlation coefficient values between gene expression and relative proportion of immune cells can indicate strong, weak or no correlation. P < 0.05 was considered a statistically significant difference by unpaired t-test.

### Immunohistochemistry and immunofluorescence

IHC [25] staining was performed with the antibody PLEK (Proteintech, 66431-1-lg, 1:500) and CD68(Abcam, ab955, 1:200) following the manufacturer’s protocol.

Apply the IHC Profiler in ImageJ 1.54d [26] to calculate the percentage contribution of High Positive, Positive, Low Positive and Negative. The staining value of PLEK and CD86 was calculated as 1-Negative (%). Immunofluorescence [27] staining of human carotid atherosclerotic plaques and intima using CD68(Abcam, ab955, 1:500) and CD163 (Abcam, ab182422, 1:1000) antibodies to investigate macrophage infiltration in plaques.

### Statistical analysis

Use unpaired t test to compare the Negative percentage contribution in GraphPad Prism 8. Other statistical analysis was performed using R 4.2.3. To compare the variations in expression between the groups, the Wilcox test was employed. The Spearman method was utilized to establish the connections between genes and immune cells. P<0.05 was considered as statistically significant difference.

## RESULTS

### Identify DEGs

**Figure 1:**
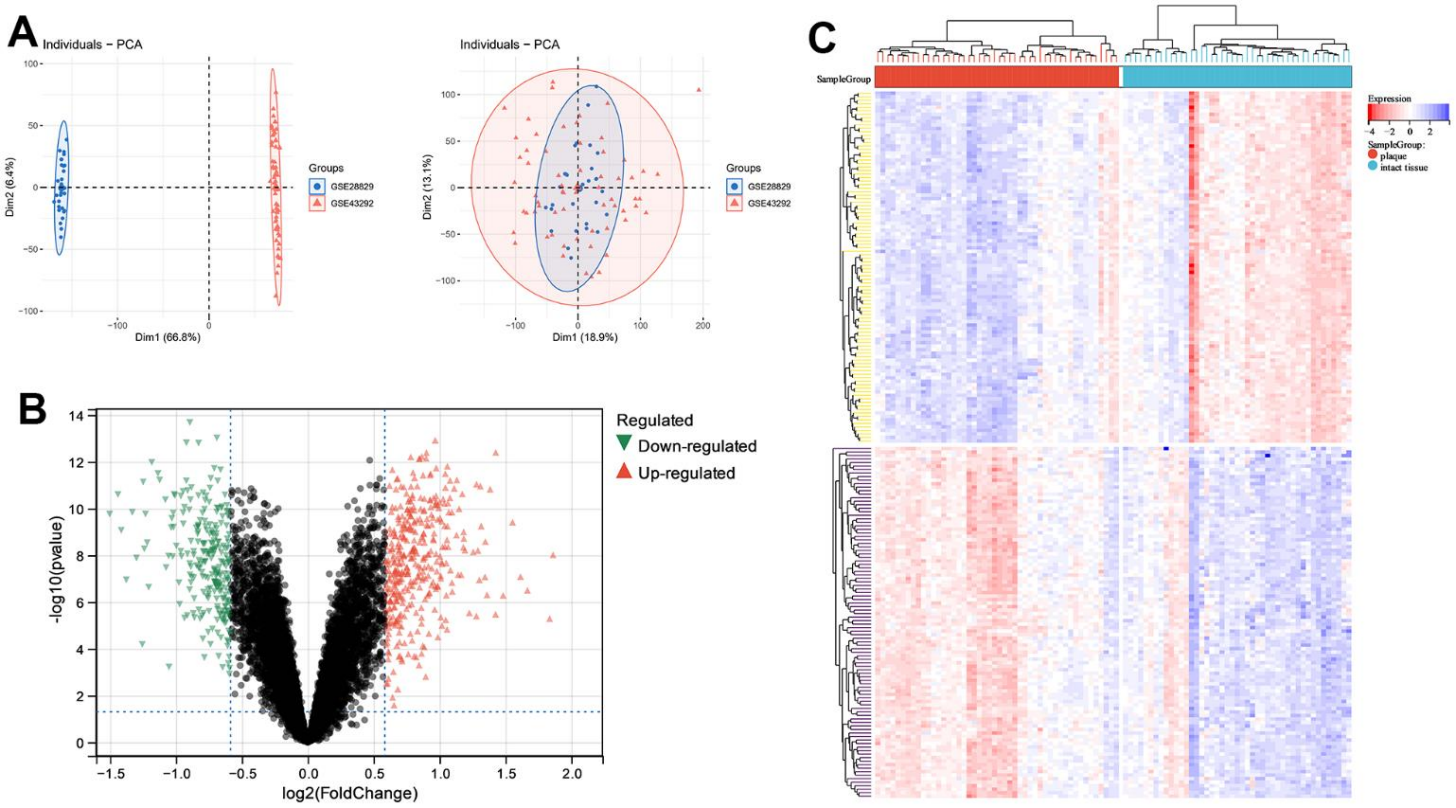
**Principle components analysis (PCA) and analysis of differentially expressed genes (DEGs) between carotid atherosclerotic plaque and intact tissue samples.** (**A**) PCA; (**B**) volcano plot; (**C**) heatmap of top 100 DEGs.

Using the gene expression matrix merged with GSE28829 and GSE43292 as the exploration dataset, PCA analysis showed the samples before and after merging (Figure 1A). Using |Fc(Fold-change)|>1.5 as the dividing line, 385 up-regulated genes and 251 down-regulated genes were shown by volcano plots (Figure 1B). The top 10 up-regulated and down-regulated DEGs of |Fc(Fold-change)| are listed in Table 1. The top 100 DEGs are represented by heatmap (Figure 1C).

**Table 1:** DEGs with top-10 |log_2_ (fold-change)| (carotid plaque/intact tissue).

**Gene symbol**	**Official full name**	**Log_2_ (fold-change)**	**Adjusted P-value**
**Up-regulated**			
**IGJ**	Immunoglobulin J Chain	2.13	7.23E-08
**MMP9**	Matrix Metallopeptidase 9	1.86	3.60E-07
**MMP12**	Matrix Metallopeptidase 12	1.84	5.26E-05
**FABP4**	Fatty Acid Binding Protein 4	1.67	5.41E-06
**CHI3L1**	Chitinase 3 Like 1	1.61	1.88E-06
**FABP5**	Fatty Acid Binding Protein 5	1.55	3.48E-08
**CD36**	CD36 Molecule	1.48	8.17E-06
**CCR1**	C-C Motif Chemokine Receptor 1	1.43	1.03E-09
**MMP7**	Matrix Metallopeptidase 7	1.43	3.72E-05
**CD52**	CD52 Molecule	1.38	4.80E-09
**Down-regulated**			
**CASQ2**	Calsequestrin 2	-1.50	1.90E-08
**CNTN4**	Contactin 4	-1.44	6.55E-09
**MYOCD**	Myocardin	-1.41	5.97E-08
**TPH1**	Tryptophan Hydroxylase 1	-1.38	2.43E-06
**PLD5**	Phospholipase D Family Member 5	-1.33	1.68E-08
**HAND2-AS1**	HAND2 Antisense RNA 1	-1.30	4.50E-07
**ITLN1**	Intelectin 1	-1.26	4.42E-04
**ACADL**	Acyl-CoA Dehydrogenase Long Chain	-1.25	3.59E-09
**CNTN3**	Contactin 3	-1.24	2.34E-07
**FHL5**	Four And A Half LIM Domains 5	-1.23	1.90E-08

### Functional enrichment analysis

**Figure 2:**
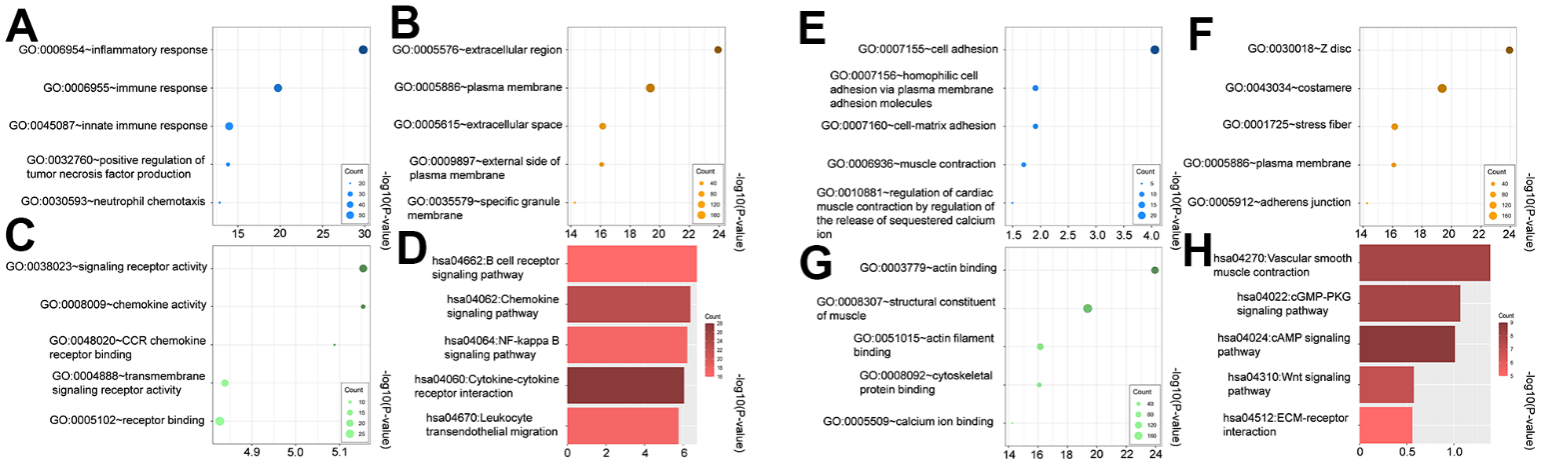
Gene ontology (GO) and kyoto encyclopedia of genes and genomes (KEGG) of upregulated (**A**–**D**) and downregulated DEGs (**E**–**H**). (**A**, **E**) biological process (BP) analysis, (**B**, **F**) cellular components (CC) analysis, (**C**, **G**) molecular function (MF) analysis, (**D**, **H**) KEGG pathway analysis.

Gene ontology including Biological Function (BP), Cell Component (CC), and Molecular Function (MF) was performed for both upregulated and downregulated genes. The GO terms that were significantly enriched after adjusting the P-value are displayed in Figure 2A–2C, 2E–2G. During the analysis of BP enrichment, the genes that were upregulated primarily participated in the inflammatory and immune responses, whereas the downregulated genes were associated with cell adhesion and homophilic cell adhesion through adhesion molecules on the plasma membrane. The CC term that showed the highest upregulation was 'extracellular region', whereas 'Z disc' exhibited the most significant downregulation. The molecular function terms indicated an increase in the activity of signaling receptors and chemokines. On the other hand, the molecule function showed a decrease in the expression of actin binding and structural constituent of muscle.

Furthermore, we performed KEGG analysis to demonstrate the pathway of differentially expressed genes (Figure 2D). Significant enrichment was observed in the upregulated pathways, including the B cell receptor, chemokine, and NF-kappa B signaling pathway. Not surprisingly, the downregulated genes exhibited enrichment in the contraction of vascular smooth muscle, as well as the cGMP-PKG and cAMP signaling pathway (Figure 2H).

### Gene set enrichment analysis (GSEA)

**Figure 3:**
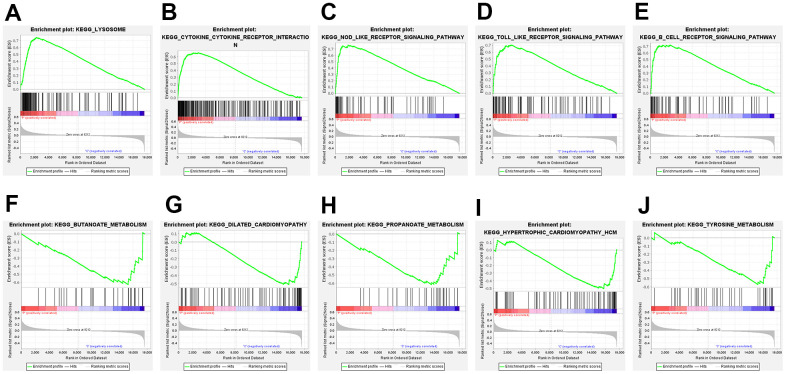
**Gene set enrichment analysis (GSEA) of the top 5 upregulated and downregulated GSEA pathways.** (**A**) lysosome; (**B**) cytokine-cytokine-receptor interaction; (**C**) NOD-like-receptor signaling pathway; (**D**) TOLL-like-receptor signaling pathway; (**E**) B-cell-receptor signaling pathway; (**F**) butanoate metabolism; (**G**) dilated cardiomyopathy; (**H**) propanoate metabolism; (**I**) hypertrophic cardiomyopathy HCM; (**J**) tyrosine metabolism.

GSEA, a computational method, evaluates the statistical significance of a priori customized gene sets, in contrast to traditional enrichment analysis that only considers a single gene. The top five pathways up- and down-regulated by GSEA are presented in Figure 3, respectively. The results showed that lysosomal, cytokine-cytokine receptor interaction, and toll-like receptor signaling pathways were upregulated, whereas butanoate metabolism and dilated cardiomyopathy were downregulated.

### Building a co-expression network

**Figure 4:**
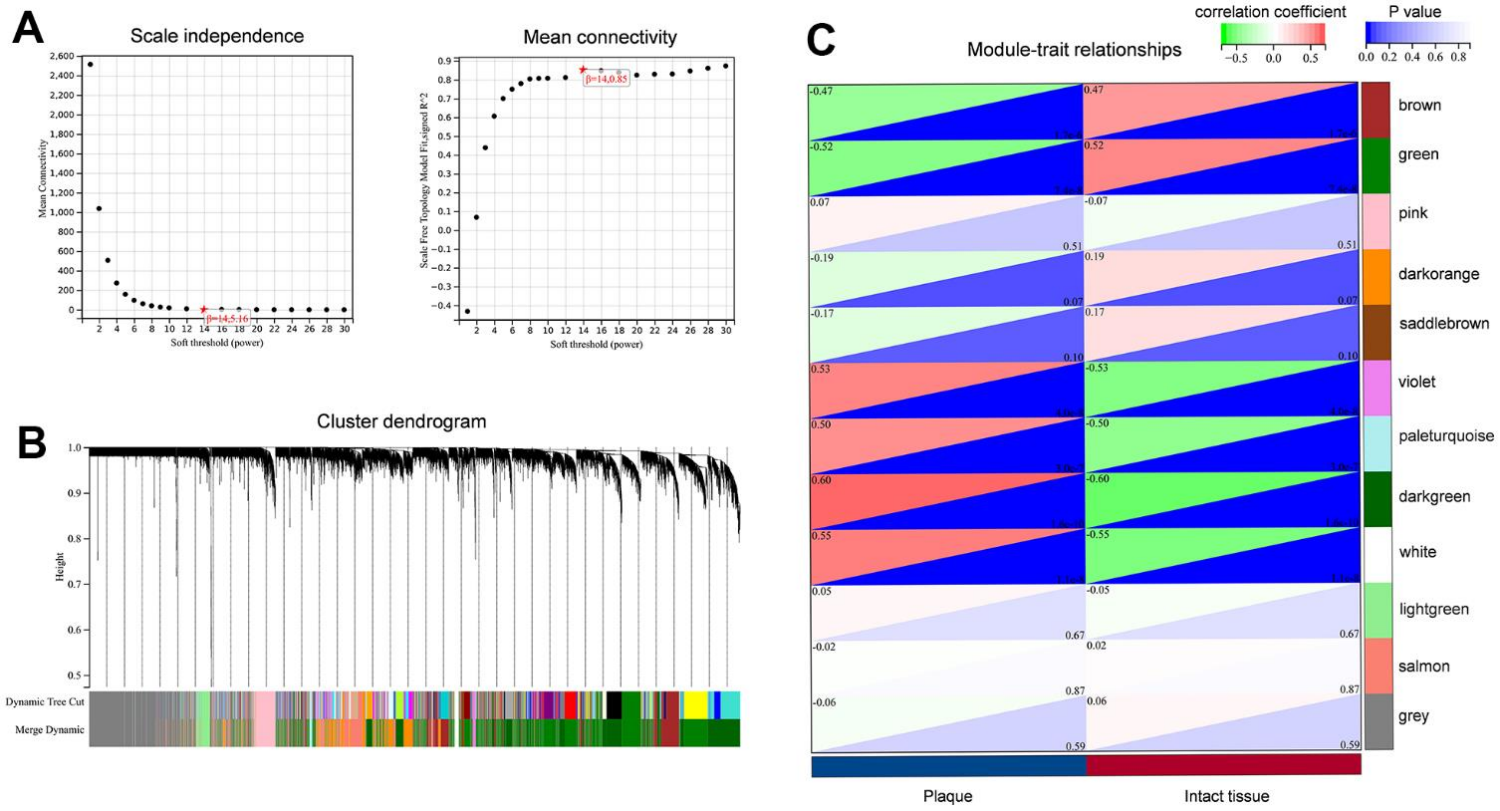
**Construction of the co-expression network for carotid atherosclerotic plaque.** (**A**) Identification of soft threshold power (β); (**B**) Clustering dendrogram to find co-expression modules; (**C**) The identification of key modules related to sample traits.

Construct a co-expression network by enrolling the combined dataset into R. There were no anomalies identified during the process of hierarchical clustering. Figure 4A depicts the construction of a scale-free network through the utilization of soft-thresholds at β=14. Twelve relevant co-expression modules were obtained (Figure 4B).

### Selection and enrichment analysis of key modules

**Figure 5:**
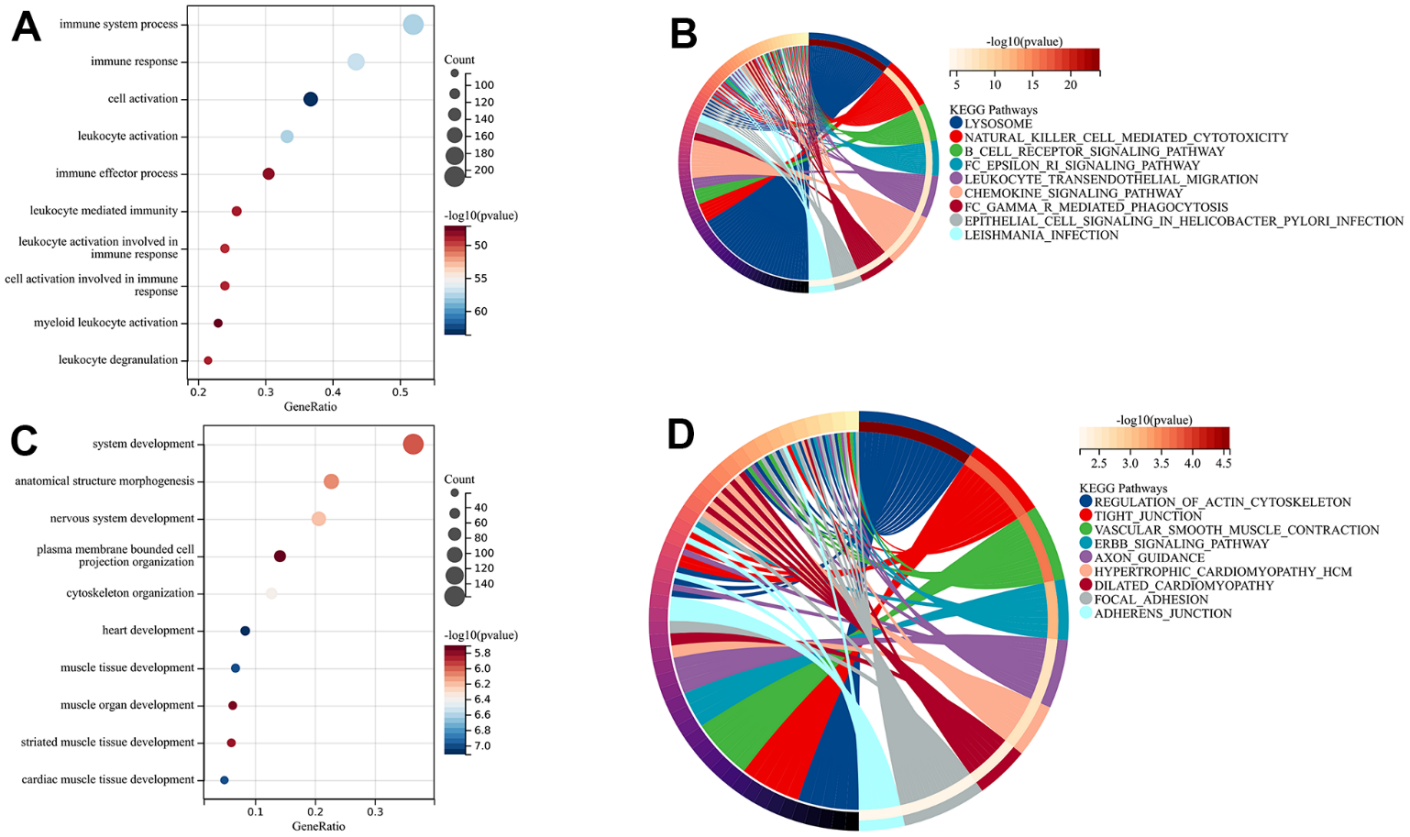
**The enrichment analysis of key modules.** (**A**, **B**) BP and KEGG analysis of the darkgreen module; (**C**, **D**) BP and KEGG analysis of green module.

In order to determine the main modules associated with sample characteristics, we developed a relationship between modules and traits (Figure 4C). We observed two co-expression modules of genes that are strongly associated with the atherosclerotic process. The darkgreen module, consisting of 2364 genes, was positively associated with sample traits in atherosclerotic plaques. In contrast, the 1886 genes found in the green module were inversely associated with advanced atherosclerotic plaques. To explore the biological functions of the key modules associated with sample traits, we also performed GO and KEGG enrichments. For the darkgreen module, BP enrichment indicated that these genes mainly participated in immune system processes, immune responses, cell activation, and leukocyte activation. KEGG analysis suggested that lysosomes might be involved in these pathways (Figure 5A, 5B). The results from GO and KEGG were highly similar to our previous enrichment analysis. The GO analysis of the green module indicated enrichment in system development, morphogenesis of anatomical structures, and development of the nervous system (Figure 5C). The KEGG findings indicated the involvement of the actin cytoskeleton, tight junctions, and regulation of vascular smooth muscle contraction, which was consistent with the results for down-regulated DEGs (Figure 5D).

### Protein-protein interactions (PPI) and screening of potential genes

**Figure 6:**
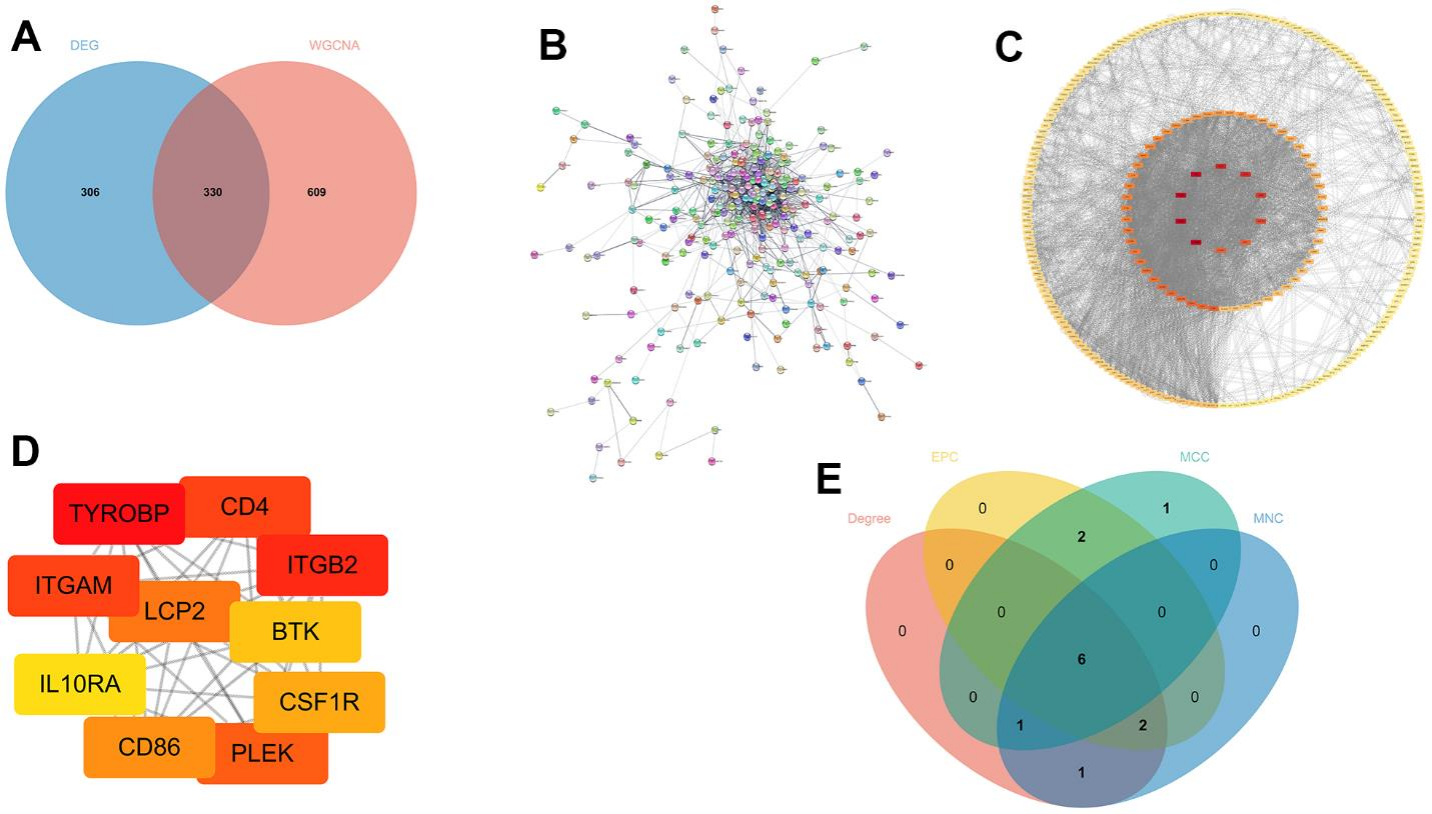
**Screening out potential genes.** (**A**) The overlapping of DEGs and key module genes from WGCNA; (**B**) Protein-protein interaction (PPI) networks; (**C**, **D**) All the genes and the top 10 genes calculated by the Degree algorithm of cytoHubba; (**E**) The overlapped hub genes from four different algorithms.

Initially, we filtered out these genes from two modules with MM>0.8, indicating that these genes exhibit a greater level of connectivity within the modules. Next, we compared the DEGs with the highly connected genes. There was an overlap of 330 genes (Figure 6A). We then imported these 330 genes into the STRING database and constructed a PPI network, ensuring a minimum required interaction score of 0.4 (Figure 6B). The results were visualized in Cytoscape and included 333 nodes and 1727 edges. The Degree algorithm utilizing the Cytoscape plugin cytohubba displays all PPI networks (Figure 6C) and the top 10 genes (Figure 6D). The top ten hub genes from the cytohubba plugin were obtained by four algorithms: MCC, MNC, Degree, and EPC. The overlapping hub genes in the four algorithms were verified by a Venn diagram (Figure 6E).

### Validation hub genes in public databases

**Figure 7:**
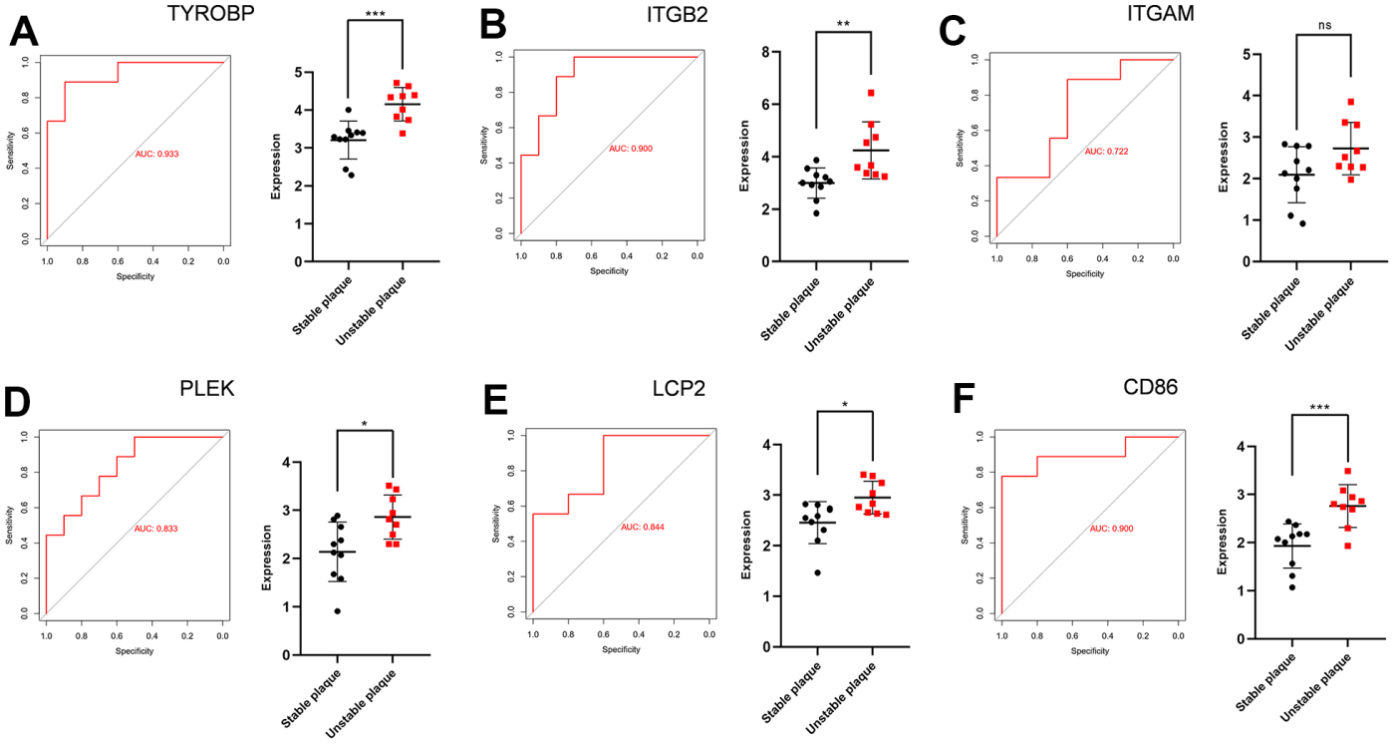
**ROC curves and statistic of expression for TYROBP, ITGB2, ITGAM, PLEK, LCP2 and CD86.** (**A**) The AUC for TYROBP was 0.933. (**B**) The AUC for ITGB2 was 0.900. (**C**) The AUC for ITGAM was 0.722. (**D**) The AUC for PLEK was 0.833. (**E**) The AUC for LCP2 was 0.844. (**F**) The AUC for CD86 was 0.900.

TYROBP (Transmembrane Immune Signaling Adaptor), ITGB2 (Integrin Subunit Beta 2), ITGAM (Integrin Subunit Alpha M), PLEK (Pleckstrin), LCP2 (Lymphocyte Cytoplasmic Protein 2), and CD86 (CD86 molecule) were the overlapping hub genes. After extracting the expression matrix profiles, the validation datasets used were GSE41571 and GSE120521. ROC analysis was conducted, revealing the potential diagnostic significance of all these essential genes. Figure 7 shows the ROC profiles and gene expression of TYROBP, ITGB2, ITGAM, PLEK, LCP2 and CD86.

### Immune cell infiltration analysis

**Figure 8:**
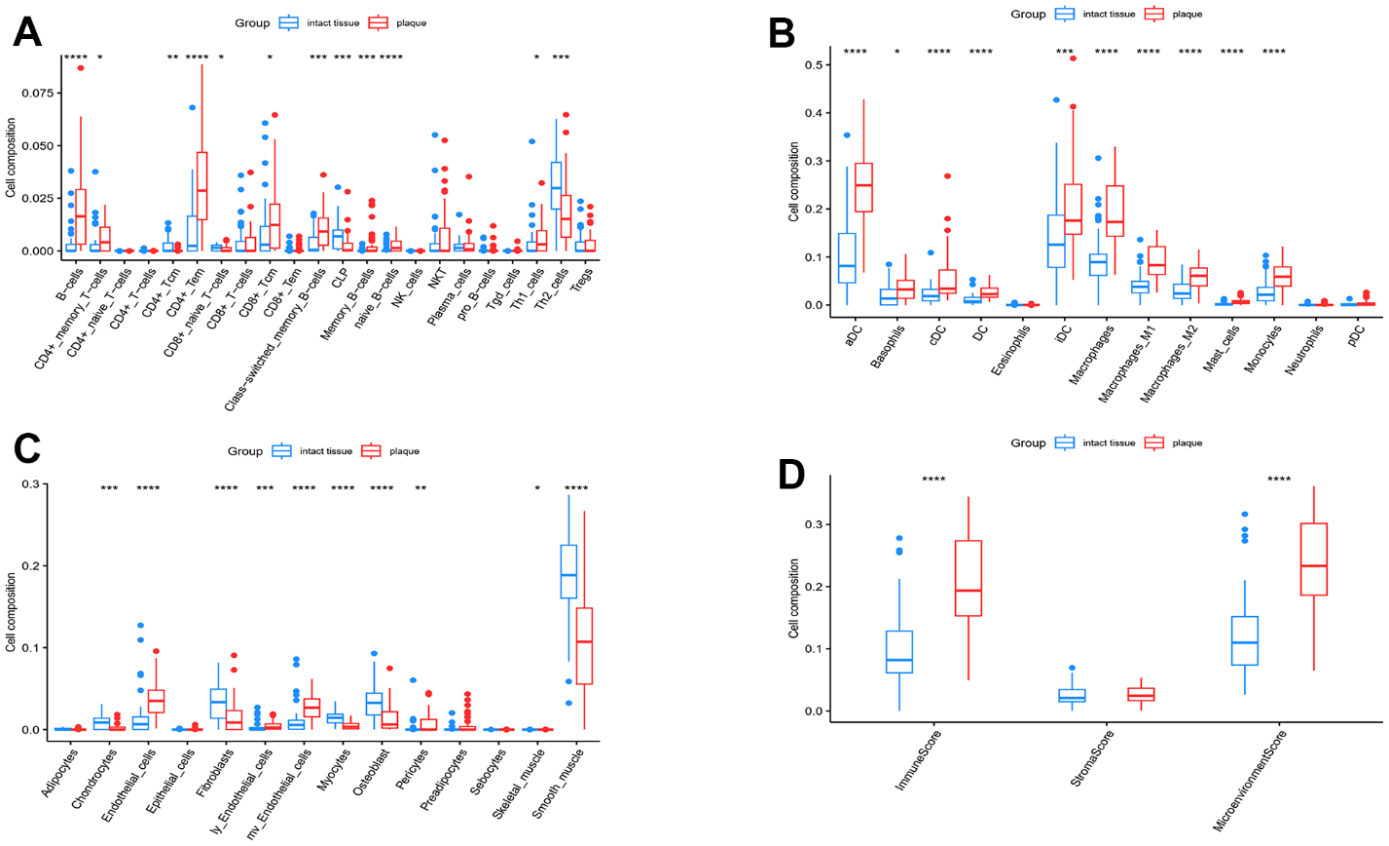
**xCell analysis.** (**A**) The enrichment scores of lymphoid cells; (**B**) Enrichment scores of myeloid cells; (**C**) Enrichment scores of stromal cells; (**D**) Total enrichment scores of the immune and stromal microenvironment. Significance level was denoted by *p-value<0.05, **p-value<0.01, ***p-value<0.001, ****p-value<0.0001.

The xCell includes 64 immune cells and stromal cells for quantitative assessment of enrichment of these cells. Figure 8 displays the types of cells and their corresponding enrichment scores. In advanced atherosclerotic plaque, the xCell scores indicated an upregulation of immunity-related cells, particularly the macrophage cells, in both lymphoid and myeloid cells (Figure 8A, 8B). On the other hand, the stromal cells exhibited a decrease in expression levels (Figure 8C). The difference in the total immune score and the microenvironmental score demonstrated immune infiltration in advanced atherosclerotic plaques (Figure 8D).

### Correlation analysis of hub genes and immune cells

**Figure 9:**
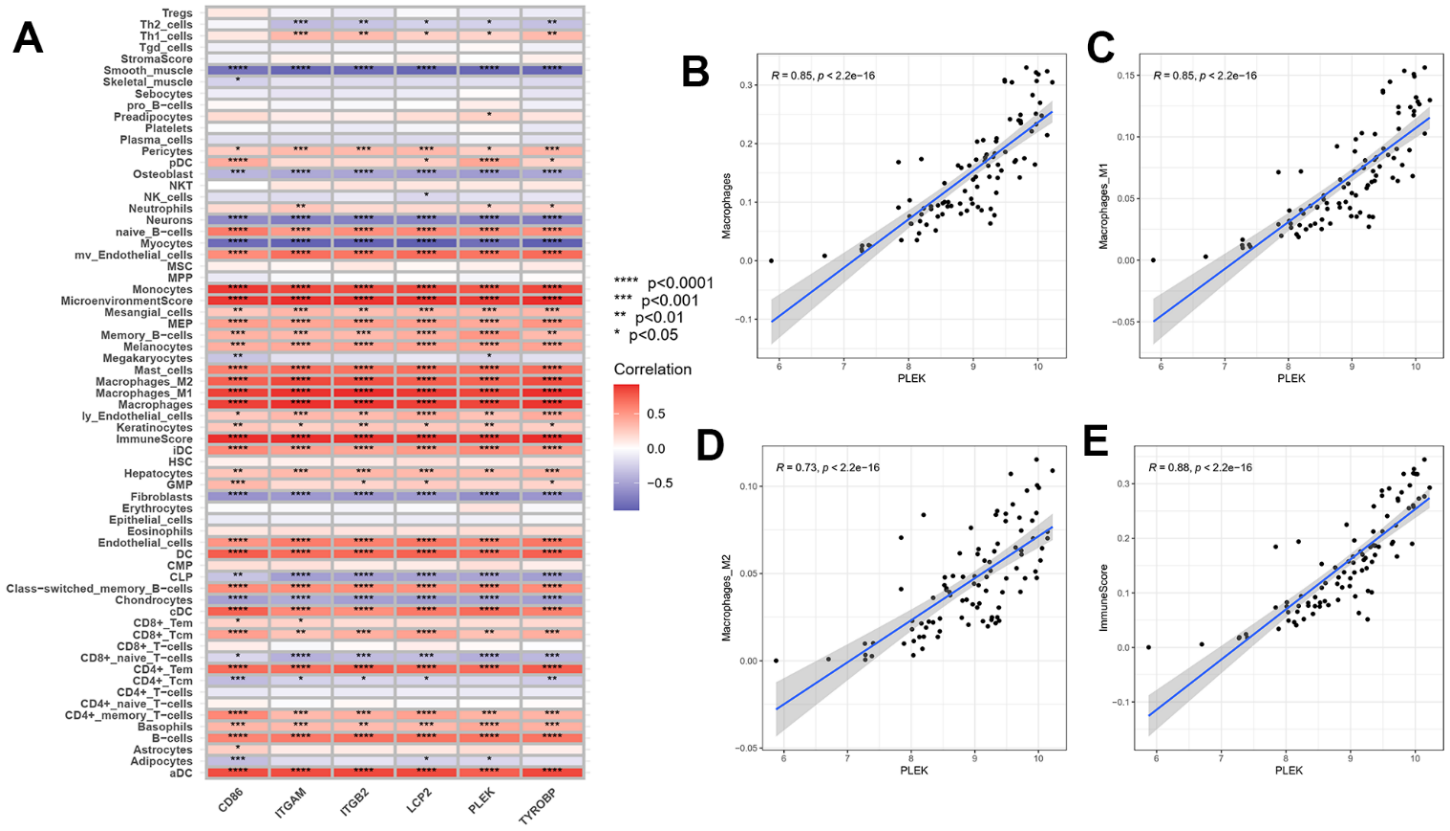
**Correlation between gene expressions and the relative percentages of 64 cell types.** (**A**) The heatmap of correlation between six hub genes and lymphoid cells, myeloid cells and stomal cells. (**B**–**E**) Scatterplots illustrate the exact relationship between the PLEK expression and the relative proportion of macrophages M0(R=0.85, p<2.2e-16), macrophages M1(R=0.85, p<2.2e-16), macrophages M2(R=0.73, p<2.2e-16) and immuneScore (R=0.88, p<2.2e-16).

To demonstrate and exhibit the connection between hub genes and immune cells in advanced plaque and intact tissue samples (Figure 9), a Spearson test was conducted for correlation analysis. The results showed that CD86, ITGAM, ITGB2, LCP2, PLEK and TYROBP are strongly positively correlated with most lymphocytes and myeloid cells, especially monocytes and macrophages. In contrast, the six hub genes are negatively correlated with most stromal cells, such as neurons, myocytes, fibroblasts and chondrocytes. As shown, PLEK has a close correlation with macrophages (R=0.85, p<2.2e-16), macrophages M1(R=0.85, p<2.2e-16), macrophages M2(R=0.73, p<2.2e-16).

### IHC validation of PLEK importance in carotid atherosclerotic plaque

**Figure 10:**
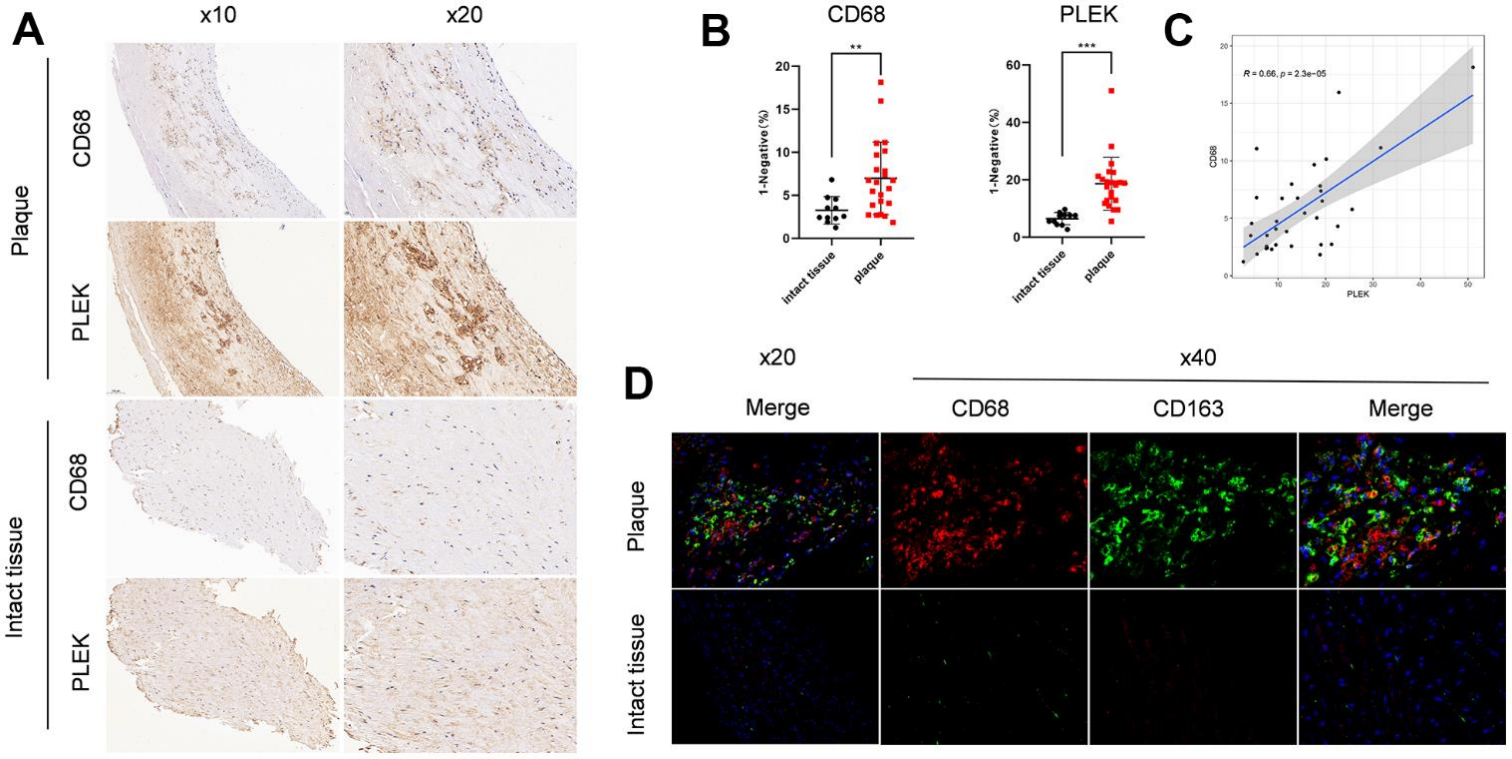
**Validation of PLEK importance in carotid atherosclerotic plaque.** (**A**) Staining images of PLEK protein expression in carotid atherosclerotic plaque and intact tissue samples. (**B**) Statistic of PLEK protein expression in carotid atherosclerotic plaque and intact tissue samples. (**C**) The scatterplot of correlation between PLEK and CD68. (**D**) Macrophage infiltration of plaque versus intact tissue by immunofluorescence.

We then identified the PLEK and CD68 expression level in 34 samples, including 23 carotid atherosclerotic plaques and 11 intact tissue samples. IHC experiment validated that carotid plaque showed higher expression of PLEK and CD68 compared to intact tissue samples (Figure 10A, 10B). Besides, we validated that PLEK has a close correlation with CD68(R=0.66, p=2.3e-05) (Figure 10C). Compared with intact tissue, Immunofluorescence staining showed increased infiltration of both CD68+ macrophages and CD163+ macrophages in carotid atherosclerotic plaques (Figure 10D).

## DISCUSSION

By combining the datasets GSE28829 and GSE43292, we identified a total of 385 genes that were upregulated and 251 genes that were downregulated in plaque samples during our study. DEGs screening in previous research has been conducted using either the microarray dataset GSE28829 or GSE43292. DEG results vary due to the use of various methods and criteria. Liu and his colleagues acquired a total of 758 genes that exhibited differential expression by utilizing GSE28829, meeting the criteria of FDR<0.05 and |log2FC|>0.58. The PPI network shows that ITGAM and ACTN2 have the greatest degree [28]. Guiming Wang et al. screened 513 upregulated genes and 373 downregulated genes. The PPI network constructed with these DEGs included 35 key nodes with degrees≥20, among which SYK, LYN and PIK3CG were the three highest [29]. Julong Guo et al. analyzed GSE41571, GSE120521, E-MTAB-2055 and one non-coding RNA dataset (GSE111794) to discover genetic molecules linked to histologically unstable carotid atherosclerotic plaques and found 10 hub genes. Among them, upregulated genes included HCK, C1QC, CD14, FCER1G, LCP1 and RAC2, while TPM1, MYH10, PLS3 and FMOD were found to be downregulated [30].

Function analysis involved genes that were upregulated and downregulated, respectively. In the biological process of gene ontology, “inflammatory response” and “immune response” were found to be enriched with up-regulated genes, while the “cell adhesion”, “homophilic cell adhesion via plasma membrane adhesion molecules” and “cell-matrix adhesion” were involved in down-regulation of genes. KEGG pathway analysis further indicated up-regulation of genes of immunity-related pathways whereas “the vascular smooth muscle contraction” was downregulated. Extensive experimental and clinical data now confirm that atherosclerosis is a persistent inflammatory condition that typically remains stable until a disruption in the arterial surface's integrity takes place, like endothelial erosion and plaque rupture [31]. In addition, experience from genome-wide association studies, advanced *in vivo* imaging, transgenic lineage tracing mice, and clinical interventional studies suggests that both innate and adaptive immune mechanisms can accelerate or inhibit atherosclerosis [32]. The role of cell adhesion is crucial in the regulation of cell migration and proliferation, particularly for VSMC in atherosclerosis. Certain initial research has indicated that the attachment of cells played a role in maintaining the health of blood vessels and safeguarding them from intraplaque hemorrhaging [33]. Hence, the compromised attachment of vascular smooth muscle cells could potentially contribute to the advancement of plaques.

The results of GSEA analysis were the same as those of KEGG analysis. In our GSEA results, lysosomal processes were upregulated. It is indicated that advanced atherosclerotic plaques exhibit autophagy mediated by lysosomes [34]. Additionally, lysosomal enzymes played a role in the destabilization of atherosclerotic plaques, including cathepsin S [35]. Thus, upregulation of the lysosome pathway may be involved in plaque progression and lead to its eventual rupture. Interestingly, butanoate, propanoate, and tyrosine metabolism were enriched in the down-regulated pathway. Short-chain fatty acids (propionate and butyrate) have been reported to be associated with energy metabolism. For example, propionate and butyrate, the major metabolites of dietary fiber, are major products of bacterial metabolism and important sources of energy [36]. It has been previously reported that acetate, butyrate, and propionate play an important role in atherosclerosis by modulating Treg cell production and inhibiting histone deacetylases (HDACs) [37, 38]. Adhesion molecules are known to promote adhesion between leukocytes and endothelial cells. Li et al. found that butyrate and propionate reduced the expression of vascular cell adhesion molecule-1(VCAM-1) [39]. Therefore, modulation of short-chain fatty acid metabolism could serve as an innovative approach to impede the formation of atherosclerotic plaques.

We then constructed a WGCNA co-expression network to mine the set of co-expression genes associated with the sample trait “Plaque”. The results revealed that two modules (darkgreen and green) were strongly associated with advanced plaques. The biological roles and KEGG pathways of the modules aligned with the DEGs. The darkgreen module was also enriched for several other immune-related biological functions, such as “leukocyte activation”, “leukocyte mediated immunity” and KEGG pathways like “leukocyte transendothelial migration”. The process of leukocyte transendothelial migration (LTEM) plays a crucial role in initiating an inflammatory immune response and sustaining chronic inflammation. Atherosclerosis leads to unregulated movement of leukocyte and leakage of blood vessels due to the compromised protective function of the endothelium [40]. Various leukocytes, especially monocytes, transit through leaky vessels to the subendothelium, polarize into macrophages, and contribute to the development of atherosclerosis.

We used the overlapped genes between DEGs and WGCNA to establish a protein-protein interaction network. We used four different algorithms to get the top 10 genes and finally got 6 overlapped genes. To validate the differential expression of these six genes, we collected two external datasets (GSE41571 and GSE120521) that included both stable and ruptured human atherosclerotic plaques. ROC curve analysis of the six genes, TYROBP, ITGB2, ITGAM, PLEK, LCP2, CD86 was performed. The AUC was 0.933 for TYROBP, 0.900 for ITGB2, 0.722 for ITGAM, 0.833 for PLEK, 0.844 for LCP2, 0.900 for CD86. Analysis of tissue expression data on porcine atherosclerosis induced by high lipid factors indicates that TYROBP, ITGB2, and ITGAM are key genes influencing the progression of ankylosing spondylitis [41]. It has been shown that upregulation of ITGB2 synergistically affects leukocyte adhesion and migration to the vascular wall, thereby influencing the progression of ankylosing spondylitis [41]. Transcriptome sequencing analysis of ox-LDL-treated endothelial cells showed that ITGAM is a key gene that influences endothelial cell apoptosis and thus promotes atherosclerosis [42]. The PLEK gene encodes the pleckstrin protein, which serves as the primary substrate for platelets and leucocytes' protein kinase C [43]. It plays a crucial role in various processes, such as G protein-coupled receptor signaling pathway, actin cytoskeleton organization, and promoting supramolecular fiber organization. The relationship between PLEK and atherosclerosis is not yet fully understood. However, there is evidence suggesting that PLEK might have a significant impact on chronic inflammatory conditions like CVD, rheumatoid arthritis (RA), and ulcerative colitis (UC) [44]. LCP2 lymphocyte cytoplasmic protein 2) encodes an adaptor protein that is a substrate for the protein tyrosine kinase pathway activated by the T-cell antigen receptor (TCR) and is thought to play a role in TCR-mediated intracellular signaling. CD86, also known as cluster of differentiation 86, is a protein produced by the CD86 gene. It is found on antigen-presenting cells (APCs) and plays a role in delivering costimulatory signals to T cells [45]. CD86, a biomarker of M1-type macrophages, is markedly expressed in vulnerable arterial plaques.M1-type macrophages release ROS and pro-inflammatory cytokines, such as TNF-α, IL-1β, IL-6, and IL-12, which damage endothelial cells and blood vessels and promote atherosclerosis [46]. Additionally, CD86 was observed to be present on fully developed dendritic cells and certain T cells [47], but the exact mechanism remains uncertain.

Given the significant importance of inflammation in carotid atherosclerotic plaques, we proceeded to perform an ssGSEA analysis utilizing 64 different immune cell types. Unsupervised hierarchical clustering revealed the immune difference between the carotid plaque and intact tissue groups. Therefore, we concluded that the presence of immune cells contributed to the advancement of carotid atherosclerotic lesions. According to the xCell results, there was a significant alteration in the ratio of various immune cells. The majority of immune cells, including B-cells, CD4+ memory T cells, DC, macrophages, and monocytes, exhibited upregulation. On the other hand, the stromal cells like chondrocytes, fibroblasts, osteoblasts, and smooth muscle cells exhibited a decrease in expression. During our analysis of immune infiltration, we observed a substantial prevalence of macrophages, which exhibited a notable rise during the progression of atherosclerosis. The classical model of macrophage polarization describes two opposing phenotypic states: the “classical” pro-inflammatory M1 macrophage and the “alternative” anti-inflammatory M2 macrophage. Nevertheless, advancements in the functional characterization demonstrate that macrophage phenotypes are not confined to the M1 and M2 extremes. Instead, they encompass a continuous range of phenotypes linked to varying cytokine production and functional traits [48, 49]. In our research, the levels of both M1 macrophages and M2 macrophages were notably increased in the advanced plaques, and all six hub genes exhibit a strong correlation with these two types of macrophages. The true function of macrophages in carotid atherosclerosis remains unclear, and it is imperative to determine the future studies' examination of the correlation between these genes and macrophages.

We conducted a comprehensive analysis and identified six hub genes involved in carotid atherosclerotic plaques. These six hub genes, TYROBP, ITGAM, ITGB2, CD86, PLEK, LCP2 may be important biomarkers for the progression of atherosclerosis and potential treatment targets. However, there still exist some inevitable difficulties in this study. For example, thanks to a lack of atherosclerosis-related datasets, we use ruptured plaque and stable plaque datasets to validate the hub genes we get from the advanced carotid plaque and early plaque samples. Besides, for financial reasons, we only verified PLEK expression in immune-histochemistry.

## CONCLUSIONS

Through bioinformatics analysis of the microarray datasets, we identified six hub genes(TYROBP, ITGAM, ITGB2, PLEK, LCP2, CD86) that may be involved in the progression of atherosclerosis correlated with immune cells, which provides clues for us to explore the pathogenesis and therapeutic approaches of cardiovascular diseases.
